# American marten and fisher do not segregate in space and time during winter in a mixed‐forest system

**DOI:** 10.1002/ece3.5097

**Published:** 2019-04-01

**Authors:** Elizabeth Croose, Florent Bled, Nicholas L. Fowler, Dean E. Beyer Jr, Jerrold L. Belant

**Affiliations:** ^1^ Vincent Wildlife Trust Ledbury UK; ^2^ Environment and Sustainability Institute University of Exeter Penryn UK; ^3^ Warnell School of Forestry and Natural Resources University of Georgia Athens Georgia; ^4^ Camp Fire Program in Wildlife Conservation State University of New York College of Environmental Science and Forestry Syracuse New York; ^5^ Wildlife Division Michigan Department of Natural Resources Marquette Michigan

**Keywords:** coexistence, *Martes americana*, niche partitioning, *Pekania pennanti*, spatial segregation, temporal segregation

## Abstract

Understanding the mechanisms of coexistence between ecologically similar species is an important issue in ecology. Carnivore coexistence may be facilitated by spatial segregation, temporal avoidance, and differential habitat selection. American martens *Martes americana* and fishers *Pekania pennanti* are medium‐sized mustelids that occur sympatrically across portions of North America, yet mechanisms of coexistence between the two species are not fully understood. We assessed spatial and temporal partitioning in martens and fishers in the Upper Peninsula of Michigan, USA, using camera trap data collected during winter 2013–2015. To investigate spatial segregation, we used a dynamic occupancy model to estimate species’ occupancy probabilities and probabilities of persistence and colonization as a function of covariates and yearly occupancy probability for the other species. Temporal segregation was assessed by estimating diel activity overlap between species. We found weak evidence of spatial or temporal niche partitioning of martens and fishers. There was high overlap in forest cover selection, and both marten and fisher occupancy were positively correlated with deciduous forests (excluding aspen [*Populus tremuloides*]). There was strong temporal overlap (${\hat{\Delta }}_{4}=0.81$; CI = 0.79–0.82) with both species exhibiting largely crepuscular activity patterns. Co‐occurrence of martens and fishers appears to be facilitated by mechanisms not investigated in this study, such as partitioning of snow features or diet. Our results add additional insights into resource partitioning of mesocarnivores, but further research is required to enhance our understanding of mechanisms that facilitate marten and fisher coexistence.

## 1 INTRODUCTION

Understanding how ecologically similar species coexist is an important issue in ecology (Frey, Fisher, Burton, & Volpe, 2017). Distinguishing patterns of co‐occurrence between species can identify the potential for ecological interactions (Waddle et al., 2010). Establishing the processes that drive co‐occurrence and interactions between species is key to understanding community assembly and diversity (Frey et al., 2017; HilleRisLambers, Adler, Harpole, Levine, & Mayfield, 2012) and can inform conservation decisions to maintain biodiversity (Bu et al., 2016).

Following the competitive exclusion principle, two competing species cannot coexist if they share the same ecological niche, and consequently, one species will be displaced (Gause, 1934; Hardin, 1960). Thus, for two competing species to coexist, they must occupy different ecological niches (Hardin, 1960; Hutchinson, 1959). Species coexistence can be facilitated by variation in morphology, ecology, behavior, or physiology (Brown & Wilson, 1956). For ecological and behavioral mechanisms, there are three major niche dimensions along which differentiation may occur: space, time, and resources (Amarasekare, 2003; Schoener, 1974). First, species may spatially segregate in response to limited resources (spatial niche partitioning) (Bu et al., 2016; Schuette, Wagner, Wagner, & Creel, 2013; Wereszczuk & Zalewski, 2015). Second, species may use resources at different times to avoid competitors (temporal niche partitioning) (Harrington et al., 2009; Kronfeld‐Schor & Dayan, 2003; Schuette et al., 2013). Third, species may specialize on distinct resources (i.e., food) or respond differently to competitors (resource partitioning) (Djagoun, Kassa, Mensah, & Sinsin, 2013; Gantchoff & Belant, 2016; Monterroso, Rebelo, Alves, & Ferreras, 2016).

Carnivore coexistence can be facilitated by spatial segregation, temporal avoidance, and differential habitat selection, which have been documented in several sympatric mustelid species (see review in Manlick, Woodford, ZuckerBerg, & Pauli, 2017). American martens *Martes americana* and fishers *Pekania pennanti* are medium‐sized mustelids that occur sympatrically across portions of North America (Fisher, Anholt, Bradbury, Wheatley, & Volpe, 2013). Both species are among the most habitat‐specialized mammals in North America and typically inhabit structurally complex late‐successional forest landscape mosaics with conifer‐dominated stands and low and closed canopies (Buskirk & Powell, 1994). During European settlement of North America, martens and fishers were extirpated from large areas due to unsustainable trapping and habitat alteration, but have subsequently recovered and recolonized parts of their historical range (Laliberte & Ripple, 2004). Martens and fishers have been reintroduced into parts of the Great Lakes region, and while fishers have been successfully re‐established, the recovery of martens has been limited (Manlick, Woodford, Gilbert, Eklund, & Pauli, 2016; Williams, Gilbert, & Zollner, 2007).

An inverse relationship between marten and fisher occurrences has been reported in several populations, with marten distribution being limited by the occurrence of the larger, dominant fisher (Fisher et al., 2013; Krohn, Elowe, & Boone, 1995; Krohn, Zielinski, & Boone, 1997; Manlick et al., 2017). Co‐occurrence of the two species is reportedly facilitated primarily by spatial segregation and differential habitat selection (Fisher et al., 2013; Karniski, 2014). While some studies have found that martens and fishers segregate spatially through selecting different land covers (Fisher et al., 2013; Karniski, 2014), others have found high overlap in land cover selection (McCann, 2011; Thomasma, 1996; Wright, 1999), with martens and fishers exhibiting vertical and horizontal spatial segregation (Thomasma, 1996). Martens preferentially select conifer stands (Fecske, Jenks, & Smith, 2002; Fisher et al., 2013; Godbout & Ouellet, 2010; Slauson, Zielinski, & Hayes, 2007) and, to a lesser extent, mixed conifer‐deciduous stands (Cheveau, Imbeau, Drapeau, & Belanger, 2013; Potvin, Bélanger, & Lowell, 2000), with high canopy closure and low fragmentation (Fecske et al., 2002; Wasserman, Cushman, Wallin, & Hayden, 2012), as well as using shrub and scrub cover (Potvin et al., 2000; Slauson et al., 2007). Fishers tend to select conifer habitat, using both mid‐ to late‐successional conifer stands (Powell, Buskirk, & Zielinski, 2003) and moderate and less dense conifer stands (Fisher et al., 2013), as well as some mixed forests (Karniski, 2014), deciduous stands (Powell et al., 2003), and shrub‐dominated areas (Fisher et al., 2013). Snow depth and density also influence the spatial distribution of martens and fishers, with deep snow limiting fisher distribution (Karniski, 2014; Krohn et al., 1995, 1997; Manlick et al., 2017).

Patterns of spatial segregation may also be due to interference competition (Fisher et al., 2013). Marten populations and distribution may be limited by fishers in part through direct predation (Krohn et al., 1997; McCann, Zollner, & Gilbert, 2011; Suffice, Asselin, Imbeau, Cheveau, & Drapea, 2017; Williams et al., 2007) or displacement (Krohn et al., 1995, 1997; Thomasma, 1996). Dietary overlap between martens and fishers is typically great (e.g., lagomorphs, small mammals) (Manlick et al., 2017; Raine, 1987; Zielinski & Duncan, 2004), even though differences in body mass of martens (839 g) and fishers (3,118 g) (Holling, 1992) should facilitate variation in foraging behavior and prey selection (Donadio & Buskirk, 2006; Rosenzweig, 1966). Temporal segregation between martens and fishers has been recorded; whereby, martens reduced activity when fishers were active to reduce the probability of encountering a fisher and potentially reduce mortality risk (McCann, Zollner, & Gilbert, 2017). Conversely, another study found that reintroduced sympatric marten and fisher populations in Wisconsin did not exhibit spatiotemporal segregation or habitat or dietary differentiation (Manlick et al., 2017). Consequently, high niche overlap and niche compression between martens and fishers, attributed to extensive landscape homogenization, have limited the recovery of martens in Wisconsin (Manlick et al., 2017).

We investigated mechanisms of co‐occurrence of martens and fishers during winter by examining resource partitioning using camera trap data from the Upper Peninsula of Michigan, USA. After extirpation from the Upper Peninsula in the early 20th century, martens were reintroduced to the area between the 1950s and 1990s and fishers between the 1960s and 1990s (Williams et al., 2007). A harvest season was opened for fishers in 1989 and martens in 2000, and currently, trappers are permitted to harvest one marten or fisher per season (Fawley, 2019). Martens and fishers have a relatively low population abundance in the Upper Peninsula, and marten populations have declined in recent years (Skalski et al., 2011; Michigan Department of Natural Resources, unpublished data).

We hypothesized that co‐occurrence of martens and fishers is facilitated by spatial or temporal niche segregation. First, we predicted that martens and fishers exhibit spatial segregation by selecting different habitat. Secondly, we anticipated that if martens and fishers do not exhibit spatial segregation, martens would alter temporal activity patterns to avoid fishers. Finally, we predicted that marten occupancy would be negatively correlated with fisher occupancy.

## 2 MATERIALS AND METHODS

### 2.1 Study area

We conducted the study in a 400‐km^2^ area in the Upper Peninsula of Michigan, USA, north of Michigamme Reservoir (46°15′N, 88°14′W) (for a full description of the study area, see Bled et al., 2015). Land cover comprised deciduous forests (38%), woody wetlands (29%), mixed forests (13%), conifer forests (6%), open water (4%), grassland/herbaceous (3%), developed (3%), and others (3%). Dominant tree species include sugar maple *Acer saccharum* and trembling aspen *Populus tremuloides* in upland deciduous forests, black spruce *Picea mariana* in lowland coniferous forests, and red pine *Pinus resinosa* in plantations. The study area is bordered by state and US highways with secondary roads interspersed throughout. The area is in a mid‐snowfall zone with average annual snowfall of 180 cm and average annual rainfall of 69 cm. Average temperatures range from 13°F in winter to 66°F in summer (Bled et al., 2015).

### 2.2 Study design

We divided the study area into 64 2.5‐ × 2.5‐km cells and selected a site in each cell considered suitable habitat. This study was originally designed to collect data on bobcats *Lynx rufus;* thus, habitat deemed suitable for bobcats was selected, though the design was also considered appropriate for collecting data on martens and fishers, as all three species occupy similar habitats (forested landscapes with a preference for conifer‐dominated stands [Buskirk & Powell, 1994; Fisher et al., 2013; Lovallo & Anderson, 1996; Reed et al., 2017]). At each site, we constructed a circular barrier of woody vegetation 0.7–1.0 m high with four entrances containing wire snares to obtain hair samples from any animals entering them and then baited the stations using vehicle‐ or hunter‐killed carcasses of white‐tailed deer *Odocoileus virginianus* or beaver *Castor canadensis*, wired to a central tree (Stricker et al., 2012). A commercial skunk‐based lure was applied to a tree approximately 1.7 m above ground. We monitored marten and fisher presence by installing a remote camera (Bushnell Infrared Trophy Cameras; Bushnell Outdoor Products, Overland Park, Kansas, USA) to a tree 70–100 cm above ground and positioned to detect animal activity at each station. Cameras were programmed to obtain images with a 5‐min delay between detections. Each station was visited every seven days, and camera batteries, memory cards, bait, and lure were replaced as required.

We conducted 8‐week surveys from 2 January to 26 February 2013–2015. We recorded the date and time of each image containing a marten or fisher. Final detection data comprised 3 years (primary survey periods), each with 4 secondary periods (i.e., data pooled in four 2‐week sessions per site, per year). For each species, we recorded if the species was detected during secondary period *j *in year *t* ($Y_{\mathrm{ijt}}^{M}$and $Y_{\mathrm{ijt}}^{F}$ for marten and fisher, respectively) and the number of days the camera was working during this same period (i.e., effort).

### 2.3 Covariates

We selected covariates based on previous studies of resource use and land cover selection by martens (Cheveau et al., 2013; Potvin et al., 2000; Slauson et al., 2007) and fishers (Fisher et al., 2013; Powell et al., 2003). We calculated vegetation cover and road and hydrological network densities in each cell, as well as distance from the station to the nearest water source. For vegetation land covers, we established six categories: deciduous (excluding aspen [*Populus* spp.]), evergreen, mixed shrub‐herbaceous (combining shrubs, scrubs, grassland, and herbaceous covers), wetland (combining woody and emergent herbaceous wetlands), aspen, and unsuitable (open water, urban) using the 2011 National Land Cover Data (30‐m resolution; Jin et al., 2013). We delineated the aspen land cover, separate from other deciduous species, using metadata from the US Department of Agriculture and National Individual Tree Species Atlas (30‐m resolution; Ellenwood, Krist, & Romero, 2015). We obtained river, stream, and road data from Topologically Integrated Geographic Encoding and Referencing system files (US Bureau of the Census, 2010).

### 2.4 Statistical analysis

We used a dynamic occupancy model (MacKenzie, Nichols, Hines, Knutson, & Franklin, 2003) to estimate each species’ occupancy probabilities and probabilities of persistence and colonization, as a function of collected covariates and yearly occupancy probability for the other species (as estimated by a single‐season model applied independently to each year; MacKenzie et al., 2002).

Using fisher as an example, we modeled occupancy $Z_{i,t}^{F}$ in year *t* and cell *i*, following a Bernoulli distribution with mean $\psi_{i,t}^{F}$ such as:

$$Z_{i,t}^{F}∼\text{Bernoulli}\;\left(\psi_{i,t}^{F}\right)$$

With mean $\psi_{i,t}^{F}$ defined as a linear expression of the considered covariates *x_i_* (vegetation cover, road and water densities, distance to the nearest water source) on the logit scale, for *t* = 1; and conditionally on previous occupancy status, and persistence and colonization probabilities ($\varphi_{i,t}^{M}$ and $\gamma_{i,t}^{M}$, respectively), for subsequent years.

$$\left\{\begin{array}{c} \text{logit}(\psi_{i,t}^{F})=\alpha +\sum \beta x_{i};t=1\\ \psi_{i,t}^{F}=\phi_{i,t}^{F}Z_{i,t}^{F}+\gamma_{i,t}^{F}\left(1-Z_{i,t}^{F}\right);t\geq 2 \end{array}\right.$$

In turn, we expressed persistence and colonization probabilities as a function of a year effect, and marten's occupancy probability $\psi_{i,t}^{M}$ as estimated using a year‐to‐year single‐season occupancy model, following:

$$Z_{i,t}^{M}∼\text{Bernoulli}\left(\psi_{i,t}^{M}\right)$$

$$\text{logit}\left(\psi_{i,t}^{M}\right)=\alpha_{t}+\sum \beta_{t}x_{i}$$

Finally, observations *Y_ijt_* for both species were modeled following a Bernoulli distribution, conditionally on the occupancy status, such as:

$$Y_{\mathrm{ijt}}∼\text{Bernoulli}\left(p_{\mathrm{ijt}}Z_{i,t}\right)$$

With species‐specific detection probability *p_ijt_* defined for each species as follows:

$$\text{logit}\left(p_{\mathrm{ijt}}\right)=\alpha +\beta \;effort_{\mathrm{ijt}}$$

We implemented the model in R (v.3.4.0, R Core Team 2017) with the package *unmarked* (Fiske & Chandler, 2011), using function *colext *for the dynamic occupancy models and function *occu* to estimate year‐specific occupancy probabilities for single‐season models. We used backward stepwise selection using Akaike's information criterion (AIC; Akaike, 1974, Burnham & Anderson, 2002) as criteria. Goodness‐of‐fit was assessed using the MacKenzie and Bailey (2004) goodness‐of‐fit test for single‐season occupancy models extended to dynamic occupancy models using a parametric bootstrap approach, implemented in the package *AICcmodavg* (Mazerolle, 2017). We scaled and centered road density, hydrological network density, and distance to the nearest water source. The linear model structure for covariates in yearly occupancy models is presented as ~Occupancy ~ Detection, while for dynamic occupancy models, they are presented as ~First‐year occupancy ~ Colonization ~ Persistence ~ Detection.

### 2.5 Temporal partitioning

We assessed temporal overlap of martens and fishers using package “overlap” (version 0.2.6) (Ridout & Linkie, 2009) implemented in R. Package “overlap” estimates species diel activity as a probability density function and measures the degree of overlap between two densities for a pair of species, thus estimating similarity of activity. We estimated marten and fisher diel activity patterns nonparametrically using kernel density estimation to measure the coefficient of overlap, $\hat{\Delta }$, which ranges from 0 (no overlap) to 1 (complete overlap) (Ridout & Linkie, 2009). We used the overlap estimator ${\hat{\Delta }}_{4}$ as recommended for large sample sizes (Meredith & Ridout, 2016) and obtained 95% confidence intervals of the overlap by bootstrapping 1,000 samples.

### 2.6 Ethics statement

This research was conducted with approval from the Mississippi State University Institutional Animal Care and Use Committee, protocol 09–004, and followed conditions of the Michigan Department of Natural Resources Scientific Collector Permit 1376.

## 3 RESULTS

Over the course of our survey, we detected martens at least once in 33 of 64 cells and fishers in 38. The two species were detected in the same cell during the same secondary period 50 times (out of 750 occasions). We detected neither species during 510 occasions. Marten and fisher were each detected alone (in one cell during one secondary period) 96 times. The number of detections and nondetections was similar for fishers and martens within and across years (Table 1). Detection probability, averaged over the 3‐year survey period and secondary occasions, was 0.53 (95% CI 0.14–0.64) for marten and 0.54 (95% CI 0.06–0.68) for fisher.

**Table 1:** The number of detections and nondetections (in parentheses) of martens and fishers, Upper Peninsula of Michigan, January–February 2013–2015

Species	Year 1 (2013)	Year 2 (2014)	Year 3 (2015)
Marten	45 (200)	52 (198)	49 (206)
Fisher	48 (197)	51 (199)	45 (210)

### 3.1 Spatial analysis

Fit for the dynamic occupancy model for fisher indicated no difference between simulated and fitted values (*χ* ^2 ^= 114.19, number of bootstrap samples = 5,000, *p* = 0.09). The variance inflation factor indicated slight overdispersion (c‐hat = 1.42). Fit for the dynamic occupancy model for marten indicated no difference between simulated and fitted values (*χ* ^2 ^= 85.09, number of bootstrap samples = 5,000, *p* = 0.25); however, the variance inflation factor indicated underdispersion (c‐hat = 0) and possible lack of fit. Consequently, we present results of dynamic occupancy models for fisher only.

Effort was selected in all yearly single‐season occupancy models for marten and dynamic occupancy models for fisher and was positively correlated with detection probability (Table 2).

**Table 2:** Model selection results of yearly marten occupancy probabilities, Upper Peninsula of Michigan, January–February 2013–2015

Model	AIC	wAIC
Year 1 (2013)		
**~hydrology + distance + DEA** **~effort**	**183.73**	**0.43**
~hydrology + distance + DEA + Evergreen ~effort	184.29	0.33
~road + hydrology + distance + DEA + Evergreen ~effort	185.86	0.15
~road + hydrology + distance + DEA + Evergreen + Wetland ~effort	187.10	0.08
~road + hydrology + distance + DEA + Evergreen + Mixed grassland + Wetland + Aspen ~effort	191.10	0.01
Year 2 (2014)		
**~DEA + Evergreen + Wetland** **~effort**	**150.40**	**0.59**
~hydrology + DEA + Evergreen + Wetland ~effort	151.95	0.27
~hydrology + distance + DEA + Evergreen + Wetland ~effort	153.71	0.11
~road + hydrology + distance + DEA + Evergreen + Mixed grassland + Wetland ~effort	157.12	0.02
~road + hydrology + distance + DEA + Evergreen + Mixed grassland + Wetland + Aspen ~effort	159.11	0.01
Year 3 (2015)		
**~hydrology + DEA + Mixed grassland + Aspen** **~effort**	**148.95**	**0.39**
~hydrology + distance + DEA + Mixed grassland + Aspen ~effort	149.25	0.34
~hydrology + distance + DEA + Mixed grassland + Wetland + Aspen ~effort	150.60	0.17
~road + hydrology + distance + DEA + Mixed grassland + Wetland +Aspen ~effort	152.44	0.07
~road + hydrology +distance + DEA +Evergreen + Mixed grassland + Wetland + Aspen ~effort	154.24	0.03

*Note*

Bold values indicate the best‐supported models.

For marten, yearly occupancy probabilities were positively correlated with deciduous forests (excluding aspen) during all years of the study, with the presence of evergreen forests and wetlands during the second year, and with mixed grassland during the third year (Table 2). A negative correlation was observed with density of hydrological features during the first and third years, distance to nearest water during the first year, and presence of aspen during the third year, with a potential negative correlation for all these covariates.

**Figure 1:**
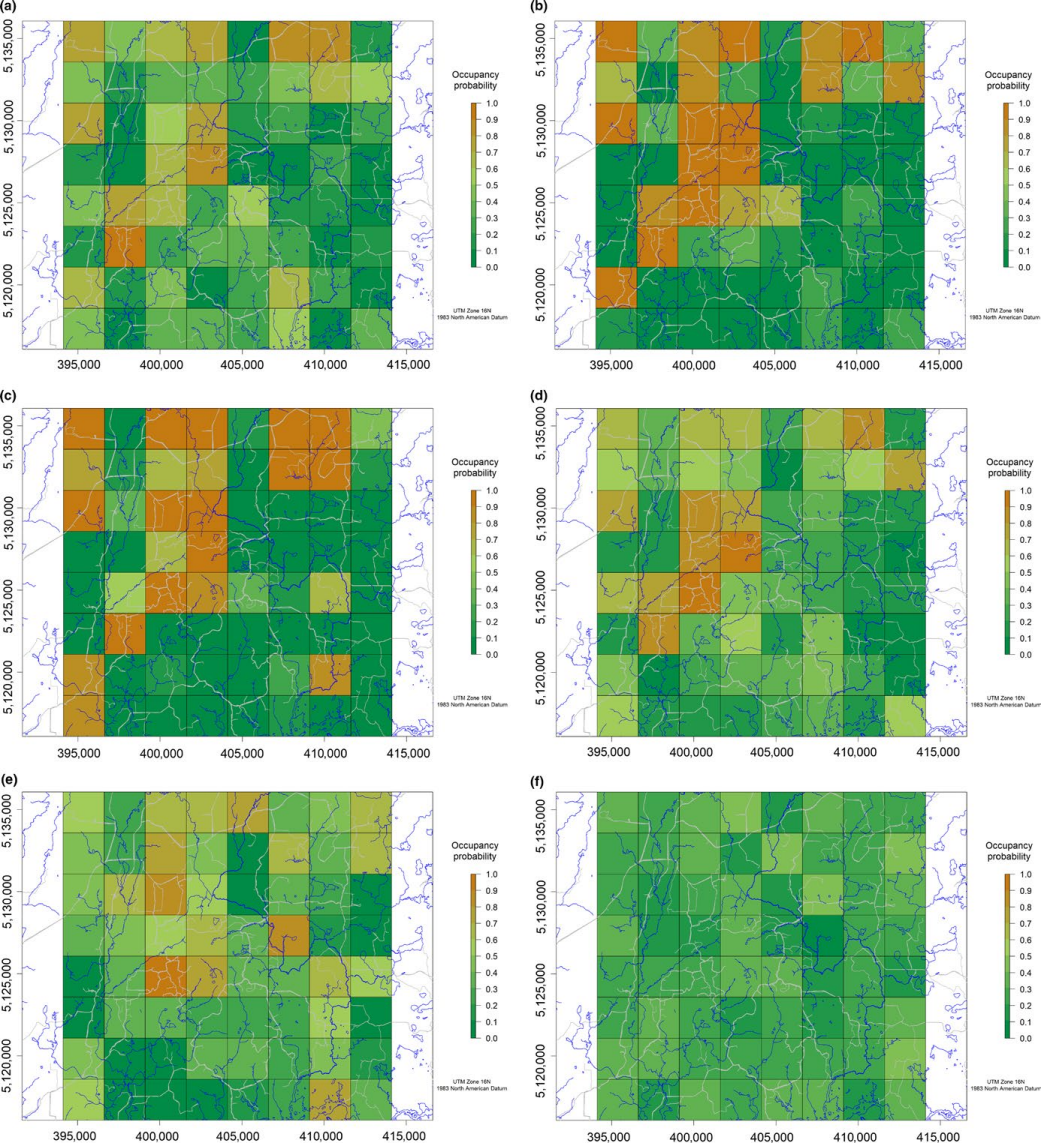
(a) Marten occupancy probability, Upper Peninsula of Michigan, January–February 2013. Roads and streams are symbolized by gray and blue lines, respectively. (b) Marten occupancy probability, Upper Peninsula of Michigan, January–February 2014. Roads and streams are symbolized by gray and blue lines, respectively. (c) Marten occupancy probability, Upper Peninsula of Michigan, January–February 2015. Roads and streams are symbolized by gray and blue lines, respectively. (d) Fisher occupancy probability, Upper Peninsula of Michigan, January–February 2013. Roads and streams are symbolized by gray and blue lines, respectively. (e) Fisher occupancy probability, Upper Peninsula of Michigan, January–February 2014. Roads and streams are symbolized by gray and blue lines, respectively. (f) Fisher occupancy probability, Upper Peninsula of Michigan, January–February 2015. Roads and streams are symbolized by gray and blue lines, respectively

Our selected dynamic model for fisher, conditioned on predicted marten yearly occupancy probabilities, indicated that initial fisher occupancy probability was positively related to road density and significantly positively correlated with deciduous forests (excluding aspen) (Table 3). Fisher colonization probability was negatively related to marten occupancy probability, which varied across years (Figure 1). None of the covariates we considered influenced persistence probability for fisher.

**Table 3:** Model selection results of dynamic occupancy probabilities for fisher as a function of marten, Upper Peninsula of Michigan, January–February 2013–2015

Model	AIC	wAIC
**~road + DEA** **~Marten + year** **~1** **~effort**	**548.12**	**0.49**
~road + DEA + Evergreen ~Marten + year ~1 ~effort	549.27	0.28
~road + hydrology + DEA + Evergreen ~Marten + year ~1 ~effort	550.56	0.14
~road + hydrology + DEA + Evergreen + Wetland ~Marten + year ~1 ~effort	552.48	0.06
~road + hydrology + DEA + Evergreen + Wetland + Aspen ~Marten + year ~1 ~effort	554.41	0.02
~road + hydrology + DEA + Evergreen + Mixed grassland + Wetland + Aspen ~Marten + year ~1 ~effort	556.21	0.01
~road + hydrology + DEA + Evergreen + Mixed grassland + Wetland + Aspen ~Marten + year ~year ~effort	558.18	0.00
~road + hydrology + DEA + Evergreen + Mixed grassland + Wetland + Aspen ~Marten + year ~Marten + year ~effort	560.17	0.00
~road + hydrology + distance + DEA +Evergreen + Mixed grassland + Wetland + Aspen ~Marten + year ~Marten + year ~effort	562.17	0.00

*Note*

Bold values indicate the best‐supported models.

### 3.2 Temporal analysis

**Figure 2:**
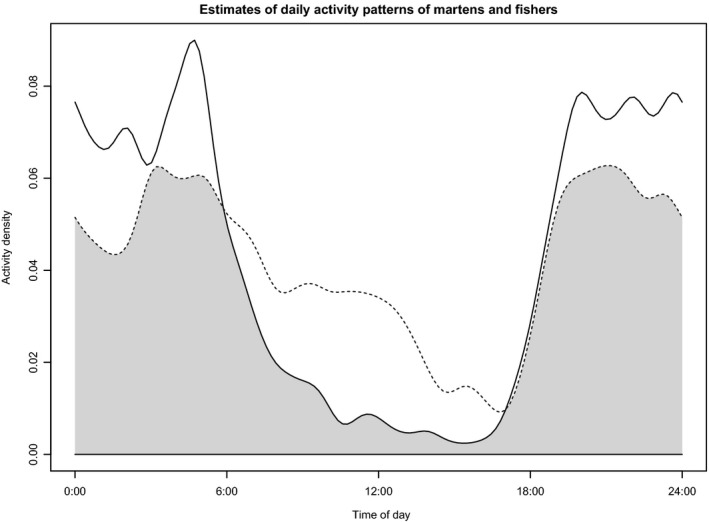
Density estimates of daily activity of martens and fishers, Upper Peninsula of Michigan, January–February 2013–2015. Area of overlap is shown as the shaded area under the minimum of the two density estimates. Fisher is displayed as a solid line and marten as a dotted line

There was a high temporal overlap in activity of martens and fishers (${\hat{\Delta }}_{4}$ = 0.81; CI = 0.79–0.82) (Figure 2). Temporal overlap increased slightly across years, from ${\hat{\Delta }}_{4}$ = 0.73 in 2013 to ${\hat{\Delta }}_{4}$ = 0.76 in 2014 to ${\hat{\Delta }}_{4}$ = 0.82 in 2015 (Table 4).

**Table 4:** Proportional overlap (95% confidence intervals) of activity of martens and fishers, Upper Peninsula of Michigan, January–February 2013–2015

Year/s	Overlap coefficient ${\hat{\Delta }}_{4}$
2013–2015	0.81 (0.79–0.82)
2013	0.73 (0.72–0.74)
2014	0.76 (0.75–0.78)
2015	0.82 (0.81–0.83)

Martens were active throughout the 24‐hr period, with peaks of activity just before dawn (06:00) and just after dusk (18:00), indicating they are mostly crepuscular. Although most activity occurred at night, martens were active during daylight hours, with least activity during late afternoon. Fishers were strongly nocturnal and active throughout the night, with peaks of activity around dawn (06:00) and dusk (18:00), tending toward crepuscular behavior. Little fisher activity occurred between 11:00 and 17:00. Greatest temporal overlap between martens and fishers occurred during the night (18:00 to 06:00) while least overlap occurred during the day, with martens more diurnal than fishers.

## 4 DISCUSSION

In contrast to our hypotheses, we found weak evidence of spatial or temporal niche partitioning of martens and fishers in the Upper Peninsula of Michigan, with martens and fishers exhibiting strong temporal overlap and high spatial overlap in forest cover.

### 4.1 Spatial partitioning

Marten and fisher occupancy were positively correlated with deciduous vegetation cover (excluding aspen), suggesting limited spatial segregation or habitat partitioning. The positive correlation between martens and deciduous cover (excluding aspen) largely contrasts with previous studies, which have found that martens select coniferous cover (Buskirk & Powell, 1994; Fisher et al., 2013; Godbout & Ouellet, 2010; Slauson et al., 2007; Thomasma, 1996). Nevertheless, in the second year of our study, marten occupancy was positively correlated with coniferous forests. Overall, martens have selected predominantly conifer stands but with a greater deciduous component in eastern North America than in western North America (Powell et al., 2003). Marten association with wetlands (during the second year of our study) supports some previous studies which have linked marten resource selection with riparian habitats, which can provide suitable sites for foraging and resting (Fecske et al., 2002; Shirk, Raphael, & Cushman, 2014). The positive correlation between marten occupancy and shrub land cover (during the third year) supports previous research documenting marten use of shrub and scrub cover (Potvin et al., 2000; Slauson et al., 2007), which they use for foraging and resting, as well as protection from predators (Godbout & Ouellet, 2010; Slauson et al., 2007).

As with martens, the positive correlation between fishers and deciduous cover (excluding aspen) is largely contrary with previous studies, which principally show a preference for coniferous and coniferous‐mixed forest, as well as shrub‐dominated areas (Fisher et al., 2013; Karniski, 2014; Linden, Fuller, Royle, & Hare, 2017; Thomasma, 1996), although fishers have been shown to use partially or entirely deciduous stands in some studies (Powell et al., 2003). Fisher occupancy was also positively related to road density, in contrast with previous studies which have shown fisher occupancy to be negatively associated with road density (Linden et al., 2017).

The contrast between our results compared with previous studies may be attributed to differences in the resolution at which data were collected. Patterns of resource selection vary across different spatial scales (Smith et al., 2013), and accordingly, spatial resolution influences the process of habitat selection (Boyce, 2006). In this study, data were collected at a relatively coarse spatial resolution, which may not reflect the fine resolution at which martens and fishers select habitat features. At coarse spatial scales, martens and fishers select coniferous forests (Fecske et al., 2002; Fisher et al., 2013), with martens also selecting mixed coniferous–deciduous forests (Cheveau et al., 2013), deciduous forests (this study), riparian habitats (Fecske et al., 2002; this study), and shrub cover (this study). At coarse spatial scales, fishers select for mixed forests (Karniski, 2014), deciduous forests (Powell et al., 2003; this study), and shrub cover (Fisher et al., 2013). At fine spatial scales, martens and fishers also select conifer‐dominated stands (Shirk et al., 2014; Thomasma, 1996), but fine‐scale resource selection by martens includes high canopy and lateral cover including dense shrub, coarse woody debris, and slash piles (Godbout & Ouellet, 2010; Potvin et al., 2000; Shirk et al., 2014), as well as riparian habitats (Shirk et al., 2014). Fine‐scale selection of fishers can include lowland coniferous forest (Powell, 1994) and high canopy cover forest (Sauder & Rachlow, 2015).

The high overlap in forest cover type selection by martens and fishers in our study has been demonstrated in other studies (McCann, 2011; Thomasma, 1996; Wright, 1999). In these cases, martens and fishers may exhibit vertical and horizontal spatial segregation to facilitate coexistence (Thomasma, 1996). Notably, it has been hypothesized that vertical and horizontal forest structure, and associated prey availability, is more important than cover type, stand composition, or age in determining marten and fisher habitat selection (Buskirk & Powell, 1994; Thomasma, 1996). Martens exploit subnivean spaces in the winter to hunt small mammals, reduce energetic costs while resting, and escape from predators (Buskirk & Powell, 1994; Sherburne & Bissonette, 1994). Conversely, subnivean access does not seem important to fishers, which mostly forage for prey above the snow (Buskirk & Powell, 1994). It has been hypothesized that the ability of martens to exploit subnivean habitats may be advantageous as it allows them to access a prey resource not available to fishers, and thus facilitates coexistence between the two species during the winter (Thomasma, 1996). This mechanism could be contributing to marten and fisher coexistence in the Upper Peninsula of Michigan and enabling both species to use the same forest cover type.

In this study, fisher colonization probability was negatively related to marten occupancy probability, supporting spatial segregation between these species. Previous studies have found an inverse relationship between marten and fisher occurrences (Fisher et al., 2013; Krohn et al., 1995,1997; Manlick et al., 2017). In a study in Alberta, Fisher et al. (2013) determined that the probability of occurrence of each species was negatively predicted by the occurrence of the other; consequently, the absence of one species significantly explained the occurrence of the other. Conversely, in Wisconsin, both marten and fisher were more likely to be detected when the other species was also present, and unexpectedly, marten detection probability was nearly twice as great in the presence of fishers (Manlick et al., 2017). Consequently, the presence of congeners can negatively or positively affect martens and fisher detections (Fisher & Bradbury, 2014).

### 4.2 Temporal partitioning

We found weak evidence for temporal segregation with strong temporal overlap between martens and fishers. Temporal segregation between martens and fishers has only been reported in one study to date (McCann et al., 2017) but has been documented in other sympatric mesocarnivores (Harrington et al., 2009; Monterroso, Alves, & Ferreras, 2014; Monterroso et al., 2016; Torretta et al., 2017). Martens and fishers exhibited largely crepuscular activity patterns consistent with other studies (Arthur & Krohn, 1991; Drew & Bissonette, 1997; Powell et al., 2003; Zielinski, Spencer, & Barrett, 1983). However, martens were active throughout the 24‐hr period including during daylight hours when fishers were rarely active, which could be a behavior used to avoid encounters with fishers. Martens may be behaviorally plastic in their activity patterns (Drew & Bissonette, 1997) and may become more diurnal in winter in cold climates to aid energy conservation (Thompson & Colgan, 1994), although this has not been the case in warmer climates where martens are primarily nocturnal in winter (Drew & Bissonette, 1997; Zielinski et al., 1983). As the data used in this study were derived from winter months only and marten and fisher activity patterns may change at different times of the year (notably, martens become more active and diurnal during the summer; Zielinski et al., 1983), studying activity patterns to compare temporal overlap throughout the year is required to enhance understanding of this topic.

### 4.3 Alternative mechanisms of co‐occurrence

Co‐occurrence of martens and fishers in our study area was facilitated by mechanisms not investigated in this study. Notably, partitioning of snow features has been consistently observed to affect patterns of spatial distribution of martens and fishers (Karniski, 2014; Krohn et al., 1997; Manlick et al., 2017; Raine, 1983). The greater footload of the larger fisher can exacerbate traveling in deep, soft snow (Leonard, 1980; Raine, 1983). Consequently, deep snow can limit fisher distribution (Karniski, 2014; Krohn et al., 1995,1997; Manlick et al., 2017; Raine, 1983) and reduce foraging opportunities that impose fitness consequences that could facilitate coexistence with martens (Manlick et al., 2017). Conversely, martens occupy areas with deep and frequent snowfall (Karniski, 2014; Krohn et al., 1997) and exhibit more subnivean behavior than fishers (Raine, 1987; Thomasma, 1996). Dietary segregation may also facilitate co‐occurrence, although dietary overlap between the two species is typically high, with limited evidence of dietary niche partitioning (Manlick et al., 2017; Zielinski & Duncan, 2004). Coexistence of mesocarnivores with high dietary overlap appears to have been facilitated by fine dietary segregation (i.e., different proportions of prey items) (Gantchoff & Belant, 2016), which may be the case for martens and fishers; this could be investigated through scat analysis. Notably, porcupines (*Erethizon dorsatum*) can be important prey for fishers (Powell et al., 2003), yet rarely occur in marten diet, which may facilitate coexistence in parts of their range. Interference competition between fishers and martens through direct predation of martens by fishers (Krohn et al., 1997; McCann et al., 2011; Suffice et al., 2017; Williams et al., 2007) or displacement (Krohn et al., 1995,1997; Thomasma, 1996) can also affect distribution and spatial patterns of both species. Finally, interactions with other species in the study area, such as red fox *Vulpes vulpes*, wolf *Canis lupus*, coyote *C. latrans*, and bobcat, may influence marten and fisher distribution (Fisher et al., 2013).

The lack of evidence of spatiotemporal partitioning in this study may also be a result of the low population abundance of martens and fishers in our study area (Skalski et al., 2011; Michigan Department of Natural Resources, unpublished data). Population density can influence resource selection, with density‐dependent processes changing the availability and quality of resources (Elkin & Reid, 2010). As such, when species are at low intraspecific densities, they may not partition habitat (van Beest, McLoughlin, Wal, & Brook, 2014). Accordingly, it may be that sympatric marten and fisher populations at relatively low densities have sufficient resources such that competition over resources does not result in partitioning.

Alternatively, the lack of spatiotemporal partitioning may also support the hypothesis of niche compression, sensu Manlick et al. (2017), where high niche overlap between martens and fishers is limiting marten recovery in Wisconsin. It is possible that niche compression of martens by fishers may also occur in our study area; marten abundance in the Upper Peninsula of Michigan has declined recently (Skalski et al., 2011), and it is unknown whether competition with fishers is influencing marten abundance. To address this question, future research on resource partitioning could evaluate focal species at varying population densities. Our results provide additional insights into resource partitioning of martens and fishers, but further research emphasizing snow features and niche compression is necessary to further clarify mechanisms of marten and fisher coexistence throughout their range.

## CONFLICT OF INTERESTS

None declared.

## AUTHOR CONTRIBUTIONS

JLB and DEB conceived the idea and designed the study. FB, EC, and NLF analyzed the data. EC led the writing of the manuscript. All authors contributed critically to the manuscript and gave final approval for publication.

## Data Availability

Data used in this manuscript are available from the Dryad Digital Repository: https://doi.org/10.5061/dryad.452n6d3.
